# ASSESSMENT OF EATING BEHAVIOR AND FOOD NEOPHOBIA IN CHILDREN AND ADOLESCENTS FROM UBERABA-MG

**DOI:** 10.1590/1984-0462/2021/39/2019368

**Published:** 2020-10-28

**Authors:** Taísa Alves Silva, Maísa Tirintan Jordani, Isabela Garcia da Cunha Guimarães, Luciene Alves, Camila Bitu Moreno Braga, Sylvana de Araujo Barros Luz

**Affiliations:** a Universidade Federal do Triângulo Mineiro, Uberaba, MG, Brazil.

**Keywords:** Feeding behavior, Food preferences, Child, Adolescent, Comportamento alimentar, Preferências alimentares, Crianças, Adolescentes

## Abstract

**Objective:**

To investigate and compare the eating behavior and food neophobia of children and adolescents from different age groups, body mass index per age, and sex.

**Methods:**

This was a cross-sectional study, with a convenience sample, involving 150 children and adolescents aged 3 to 13 years, of both sexes, treated at a pediatric outpatient clinic of a teaching hospital in the municipality of Uberaba-MG, Brazil. Subscales of the Child Eating Behavior Questionnaire (CEBQ) were used to evaluate eating behavior, and the Child Food Neophobia Scale (CFNS) was used to evaluate food neophobia.

**Results:**

Higher scores were found in the subscales “food responsiveness” (p=0.015), “enjoyment of food” (p=0.002), and “emotional overeating” (p=0.009) among older children and adolescents. Younger children had higher scores in the subscales “satiety responsiveness” (p=0.004) and “slowness in eating” (p=0.001). There was a tendency toward higher scores for “food responsiveness” (p=0.005) and “emotional overeating” (p=0.013) in participants with severe obesity. There were no differences in the scale of food neophobia. Overall, food neophobia positively correlated with lack of interest in food and negatively correlated with interest in food.

**Conclusions:**

The study showed significant differences in some domains of eating behavior among children and adolescents of the sample; however, no differences were found regarding food neophobia. These results may contribute to the improvement of future interventions related to infant eating behavior and food neophobia.

## RESUMO

**Objetivo:**

Investigar e comparar o comportamento e a neofobia alimentares de crianças e adolescentes de diferentes faixas etárias, índice de massa corporal/idade e sexo.

**Métodos:**

Tratou-se de um estudo transversal, com amostra por conveniência, envolvendo 150 crianças e adolescentes com faixa etária entre 3 e 13 anos, de ambos os sexos, atendidas em um ambulatório pediátrico de um hospital escola em Uberaba, MG. Para avaliar o comportamento alimentar, utilizaram-se as subescalas do *Child Eating Behaviour Questionnaire* (CEBQ) e para a neofobia alimentar o *Child Food Neophobia Scale* (CFNS).

**Resultados:**

Verificaram-se pontuações mais altas nas subescalas “resposta à comida” (p=0,015), “prazer em comer” (p=0,002) e “sobreingestão emocional” (p=0,009) em crianças e adolescentes com idades maiores. As crianças com idades menores obtiveram pontuações mais altas nas subescalas como “resposta à saciedade” (p=0,004) e “ingestão lenta” (p=0,001). Observou-se uma tendência à maior pontuação quanto à “resposta à comida” (p=0,005) e à “sobreingestão emocional” (p=0,013) nos participantes com obesidade grave. Não houve diferenças quanto à escala de neofobia alimentar. De modo geral, a neofobia alimentar correlacionou-se positivamente com o desinteresse pela comida e negativamente com o interesse pela comida.

**Conclusões:**

O estudo indicou diferenças significantes em alguns domínios do comportamento alimentar entre as crianças e os adolescentes da amostra, porém não houve diferenças em relação à neofobia alimentar. Nesse sentido, esses resultados podem contribuir para o aprimoramento de intervenções futuras referentes ao comportamento e à neofobia alimentares infantis.

## INTRODUCTION

Eating behavior, understood as the set of cognition and feelings that interact with physiological, psychological, and external conditions and that govern actions, eating behaviors, and the act of eating,^1^ has been considered in many studies related to overweight and obesity.^2^ ^,^ ^3^ ^,^ ^4^ ^,^ ^5^ ​Regarding these issues, in 2008 the prevalence of 33.5 and 14.3% of overweight and obesity was found, respectively, in children under 10 years of age; and a prevalence of 20.5 and 4.9%, in adolescents aged between 10 and 19 years,^6^ supporting the need for studying this group.

 Eating behavior is composed of several dimensions that can be classified into groups such as “interest in food” and “lack of interest in food.”^7^ Studies observed that overweight children and adolescents have more “interest in food,” and they may be more ​responsive to food, have greater enjoyment in eating, and consume more food in the presence of emotions, which characterizes the emotional overeating.^2^ ^,^ ^5^ ^,^ ^7^ ^,^ ^8^ Furthermore, this population may be less responsive to satiety and express greater desire to drink,^2^ ^,^ ^7^ ^,^ ^8^​ thus promoting weight gain. Conversely, “lack of interest in food” was more frequently found in eutrophic and underweight children and adolescents,^5^ who showed more regulation as for satiety control, ​slowness in eating, and less food consumption in the presence of emotions, which characterizes the emotional undereating.^2^ ^,^ ^3^ ^,^ ^7^​ Another factor found in this last group was food fussiness,^5^ ^,^ ^7^​ which also expresses “lack of interest in food” and may be associated with other behavioral changes such as food neophobia.

 Food neophobia is characterized by resistance or difficulty in eating and trying new foods,^9^​ is more frequent in school-age children, and can perpetuate through adulthood.^1^ In some cases, food neophobia acts as a protective mechanism to prevent the consumption of contaminated foods, but it can limit the variety of consumed foods.^1^ ^,^ ^9^​ To assist children and adolescents who have these eating disorders, parents and caregivers use some strategies such as persuasion, coercion, bribery, reward, or even food restriction. However, studies have shown that these attitudes can have the opposite effect^​^ ^3^ ^,^ ^10^ ^,^ ^1^ or even worse maladjusted eating behaviors, such as appetite regulation, hunger, and satiety, being able to cause changes in weight and bad relationship with food.^10^

 To date, the scientific literature states that some dimensions of eating behavior, such as satiety regulation, are innate; but, due to changes related to environment, parental attitudes, and other factors,^1^ they can be associated with different nutritional statuses^2^ ^,^ ^3^ ^,^ ^5^ and hinder the treatment of food neophobia.^10^ ^,​^ ^1^​ In addition, the development of inappropriate eating habits and behaviors of children and adolescents can lead to overweight and obesity, which are risk factors for chronic diseases that last through adulthood.^11^

Taking this into consideration, the understanding of eating behavior and food neophobia is paramount to propose actions that assist parents, caregivers, and professionals in encouraging changes in behavior, heath promotion, and, consequently, the reduction of overweight in children and adolescents. However, there is a gap in studies that assess the relationship between eating behavior and food neophobia in this population. The objective of the present study was to investigate and compare eating behavior and food neophobia between children and adolescents from different age groups, body mass index according to age (BMI-for-age), and sex.

## METHOD

This is a cross-sectional study conducted from June to November 2018 in a pediatric outpatient clinic of a teaching hospital in the city of Uberaba, State of Minas Gerais, Brazil. A convenience sampling was carried out, involving 150 children and adolescents aged from 3 to 13 years, of both sexes, who only consumed foods orally, and were accompanied by parents or caregivers who knew their daily eating habits and behaviors well enough. This population was chosen due to the increasing prevalence of overweight and obesity in this age group.^6^

Parents and caregivers completed three self-administered questionnaires in the presence of the researcher to minimize data loss, considering that the outpatient clinic serves patients from several municipalities: the Child Eating Behavior Questionnaire (CEBQ), the Child Food Neophobia Scale (CFNS), and a structured socioeconomic questionnaire in order to characterize the sample. Moreover, the weight and height of children and adolescents were measured after completing the questionnaires.

 CEBQ is a specific questionnaire applied to investigate eating behavior in children and young people according to the answers provided by their parents or guardians, focusing on behavioral determinants of obesity.^7^​ The instrument consists of 35 items whose objective is to evaluate eight subscales, namely: “satiety responsiveness” (SR), “slowness in eating” (SE), “food fussiness” (FF), “food responsiveness” (FR), “enjoyment of food” (EF), “desire to drink” (DD), “emotional overeating” (EOE), and “emotional undereating” (EUE). Responses are marked on a 5-point Likert scale that indicates the frequency with which the behavior occurs, in which 1 means “never”; 2, “rarely”; 3, “sometimes”; 4, “often”; and 5, “always.” This questionnaire has been validated in research conducted in the United Kingdom^12^​ and in Portugal,^5^ whose authors assessed children and adolescents aged 4 to 5 years and 3 to 13 years, respectively. Due to the international recognition of CEBQ^5^ ^,^ ^7^ ^,^ ^12^ and the scarcity of questionnaires validated in Brazil that address this issue, the present study used the version translated to an European Portuguese population,^5^ replacing the word “*sumos*” (European Portuguese word for “juices”) with “*sucos*” (Brazilian Portuguese word for “juices”).

 Conversely, the CFNS is a version consisting of ten items similar to those of the Food Neophobia Scale applied to adults, but used for parents or guardians to report children’s traits of food neophobia and appetite to try new foods.^13^ The responses vary from 1 to 5 - “Strongly agree” (1), “Agree” (2), “Neither agree nor disagree” (3), “Disagree” (4), and “Strongly disagree” (5) - and the total score ranges from 10 to 50 points, in which lower values indicate higher levels of food neophobia. This questionnaire has been widely used to measure food neophobia among children of different ages and has shown good internal consistency, with Cronbach’s Alpha coefficients ranging between 0.81^14^ and 0.91.^15^​ The European Portuguese version of the CFNS was developed considering the original scale and adaptations of secondary items introduced in the Australian version,^15^ and the final version was tested with a small sample of parents of children aged 2 to 6 years to confirm the clarity of the items and instructions for completing the instrument.^16^​

 The socioeconomic questionnaire consisted of questions about the number of family members living in the same residence (“up to 3 family members,” “more than 3 family members”), area of residence (“rural,” “urban”), residence status (“own,” “rent”), education of parents or caregivers and family income (“less than 1 minimum wage,” “1‒1.9 minimum wages,” “2‒3 minimum wages,” “more than 3 minimum wages,” and “do not know”), using the minimum wage value as a reference (BRL 954.00 equivalent to USD 247.80, values concerning the year 2018). Children and adolescents were classified in groups, such as age groups (<6, 6‒7, 8‒9, 10‒13 years old), sex, and BMI-for-age, for later comparison between CEBQ and CFNS scores.

It is worth noting that the previously trained research team collected anthropometric measurements, in which weight (kg) was measured on a digital scale (BALMAK^®^, Santa Bárbara d’Oeste, Brazil) with a capacity of up to 200 kg and precision of 0.1 kg; and height (cm) was measured in a stadiometer with a 0.1-cm graduation; BMI was subsequently calculated (BMI=weight(kg)/height (m)^2^). To assess the nutritional status, the BMI-for-age z-score was used, which was classified according to the growth curves proposed by the World Health Organization (WHO)^17^ in the following categories: severe thinness, thinness, normal weight, risk of overweight, overweight, obesity, and severe obesity. To calculate and classify the z-score, the WHO Anthro^®^ and AnthroPlus^®^ software were used (Geneva, Switzerland).^17^

 Data processing and analysis were performed using the Statistical Package for the Social Sciences (SPSS) software, version 22.0 (IBM Corporation, New York, United States of America). The relative frequencies of the following variables were presented: sex, age, socioeconomic characterization, and BMI-for-age classification. Normality of data was assessed by the Kolmogorov-Smirnov test. The scores of the CEBQ subscales and the final CFNS score were indicated as mean and standard deviation. The Kruskal-Wallis test was used to compare the scores of the questionnaires between the age groups and the BMI-for-age, and the Mann-Whitney test was used to compare the scores between sex. For correlations between the CEBQ subscales and the final CFNS score, the Pearson’s coefficient was employed. The adopted significance level was 5% (p<0.05).

 The project was approved by the Research Ethics Committee of Universidade Federal do Triângulo Mineiro (UFTM) under protocol No. 2,625,851, and all those responsible for children and adolescents signed an informed consent form.

## RESULTS

A total of 150 children and adolescents participated in the study, and 64.0% (n=96) were male. Age varied between 3 and 13 years old, with a mean of 7.7±2.9 years, distributed into four groups with approximately the same sample size: <6 years (n=41), 6-7 years (n=32), 8-9 years (n=36), and ≥10 years (n=41). The average age of parents and caregivers was 35.8±9.9 years, and the education level that prevailed was high school (40.0%; n=60), followed by some elementary school (20.7%; n=31).

Regarding the socioeconomic characterization of the sample, most (73.3%; n=110) had more than three family members living in the same residence, and 96.7% (n=145) lived in an urban area. In addition, 70.0% (n=105) of the sample had their own residence, 32.7% (n=49) had a family income of 1 to 1.9 minimum wages, followed by 31.3% (n=47) with 2 to 3 minimum wages.

When analyzing the BMI-for-age of children and adolescents, based on the z-score, a prevalence of normal weight was found (63.3%; n=95), followed by 14.7% (n=22) overweight, 12.0% (n=18) obesity, 7.3% (n=11) severe obesity, and 2.7% (n=4) risk of overweight, indicating that 36.7% of the sample was overweight. No participants grouped in the thinness category were found in the sample.

The means and standard deviation of the scores of the CEBQ subscales and the CFNS, according to age group, were demonstrated in Table 1. In this comparison, significant differences were found between some CEBQ subscales regarding the interest in food, such as “food responsiveness” (p=0.015), “enjoyment of food” (p=0.002), and “emotional overeating” (p=0.009), in which a tendency for higher scores was observed between children and adolescents aged 8 to 13 years. Regarding lack of interest in food, the means of the subscale scores, such as “satiety responsiveness” (p=0.004) and “slowness in eating” (p=0.001), showed significant differences, with a tendency for higher scores between children aged ≤7 years.

**Table 1:** Mean (±standard deviation) of scores of the subscales of the Child Eating Behavior Questionnaire (CEBQ) and the Child Food Neophobia Scale (CFNS) according to age group.

	Age group (n=150)				
	<6 years (n=41)	6‒7 years (n=32)	8‒9 years (n=36)	10‒13 years (n=41)	p-value
SR	3.0 (1.1)	2.9 (1.1)	2.6 (1.1)	2.2 (0.9)	**0.004**
SE	3.1 (1.3)	2.9 (1.3)	2.6 (1.4)	2.0 (1.1)	**0.001**
FF	3.1 (1.0)	3.1 (1.1)	3.2 (1.2)	3.3 (1.2)	0.837
EUE	2.9 (1.2)	2.4 (1.2)	2.5 (1.1)	2.4 (1.1)	0.210
FR	2.6 (1.2)	2.9 (1.4)	3.4 (1.4)	3.4 (1.2)	**0.015**
EF	3.4 (1.2)	3.7 (1.1)	4.0 (1.1)	4.4 (0.6)	**0.002**
EOE	2.1 (1.0)	2.1 (1.0)	3.0 (1.3)	2.3 (1.1)	**0.009**
DD	3.9 (1.4)	3.8 (1.6)	3.4 (1.5)	3.5 (1.6)	0.511
CFNS	28.2 (6.1)	29.8 (5.6)	28.5 (6.3)	28.2 (5.5)	0.557

Subscales of the Child Eating Behavior Questionnaire - SR: satiety responsiveness; SE: slowness in eating; FF: food fussiness; EUE: emotional undereating; FR: food responsiveness; EF: enjoyment of food; EOE: emotional overeating; DD: desire to drink. CFNS: Child Food Neophobia Scale.

As for scores related to BMI-for-age classifications, significant differences were observed only in the CEBQ subscales “emotional undereating” (p=0.041), “food responsiveness” (p=0.005), and “emotional overeating” (p=0.013) (Table 2). In these last two subscales, related to interest in food, a higher score was verified in participants with severe obesity when compared with participants with normal weight.

**Table 2:** Mean (±standard deviation) of scores of the subscales of the Child Eating Behavior Questionnaire (CEBQ) and the Child Food Neophobia Scale (CFNS) according to body mass index per age (BMI-for-age).

	BMI-for-age (n=150)					
	Normal weight (n=95)	Risk of overweight (n=4)	Overweight (n=22)	Obesity (n=18)	Severe obesity (n=11)	p-value
SR	2.7 (1.1)	3.8 (0.9)	2.7 (1.1)	2.5 (1.0)	2.4 (1.0)	0.227
SE	2.8 (1.3)	3.1 (1.6)	2.2 (1.2)	2.5 (1.3)	2.0 (1.2)	0.136
FF	3.0 (1.1)	3.4 (1.0)	3.3 (1.8)	3.5 (1.3)	3.6 (1.1)	0.285
EUE	2.4 (1.1)	4.2 (0.7)	2.7 (1.3)	2.8 (1.0)	2.1 (0.9)	**0.041**
FR	2.8 (1.3)	3.8 (1.0)	3.0 (1.5)	3.6 (1.3)	4.2 (0.9)	**0.005**
EF	3.8 (1.1)	3.7 (1.6)	3.7 (1.1)	4.1 (1.0)	4.4 (0.6)	0.436
EOE	2.1 (1.0)	2.7 (1.1)	2.3 (1.2)	2.9 (1.2)	3.4 (1.5)	**0.013**
DD	3.6 (1.5)	3.3 (1.6)	4.0 (1.4)	3.7 (1.6)	3.0 (1.8)	0.531
CFNS	29.1 (6.0)	25.8 (2.6)	27.6 (5.4)	28.7 (5.1)	27.2 (7.6)	0.581

Subscales of the Child Eating Behavior Questionnaire - SR: satiety responsiveness; SE: slowness in eating; FF: food fussiness; EUE: emotional undereating; FR: food responsiveness; EF: enjoyment of food; EOE: emotional overeating; DD: desire to drink. CFNS: Child Food Neophobia Scale.

There were no significant differences in the scores between sexes, but a tendency for higher scores in the subscale “emotional overeating” was observed in males (Table 3).

**Table 3:** Mean (±standard deviation) of scores of the subscales of the Child Eating Behavior Questionnaire (CEBQ) and the Child Food Neophobia Scale (CFNS) according to sex.

	Sex (n=150)		
	Male (n=96)	Female (n=54)	p-value
Satiety responsiveness (SR)*	2.6 (1.0)	2.9 (1.1)	0.123
Slowness in eating (SE)*	2.6 (1.3)	2.6 (1.3)	0.858
Food Fussiness (FF)*	3.2 (1.1)	3.2 (1.1)	0.834
Emotional undereating (EUE)*	2.6 (1.1)	2.4 (1.2)	0.206
Food responsiveness (FR)*	3.2 (1.3)	2.8 (1.3)	0.078
Enjoyment of food (EF)*	3.9 (1.0)	3.8 (1.1)	0.423
Emotional overeating (EOE)*	2.5 (1.2)	2.1 (1.1)	0.055
Desire to drink (DD)*	3.8 (1.5)	3.4 (1.6)	0.232
Food neophobia (CFNS)	28.7 (5.7)	28.4 (6.2)	0.545

* Subscales of the Child Eating Behavior Questionnaire

Table 4 shows the correlations of the mean scores of each of the CEBQ subscales between each other and with the total score of the CFNS. The subscale “satiety responsiveness” positively correlated with the scores of “slowness in eating” (p=0.000), “food fussiness” (p=0.001), and “emotional undereating” (p=0.006); and negatively correlated with “food responsiveness”(p=0.000), “enjoyment of food” (p=0.000), and CFNS scores (p=0.000).

**Table 4:** Correlation between subscales of the Child Eating Behavior Questionnaire (CEBQ) and with total scores of the Child Food Neophobia Scale (CFNS).

		SR	SE	FF	EUE	FR	EF	EOE	DD	CFNS
SR	r	1	0.364	0.258	0.221	-0.449	-0.607	-0.128	-0.008	-0.336
	p-value		**<0.001**	**0.001**	**0.006**	**0.000**	**<0.001**	0.118	0.921	**<0.001**
SE	r		1	0.075	0.166	-0.254	-0.330	-0.114	0.000	-0.118
	p-value			0.360	**0.042**	**0.002**	**<0.001**	0.166	0.998	0.151
FF	r			1	0.014	0.010	-0.205	0.077	0.045	-0.722
	p-value				0.863	0.902	**0.012**	0.348	0.584	**<0.001**
EUE	r				1	-0.010	0.004	0.234	0.138	-0.185
	p-value					0.905	0.964	**0.004**	0.092	**0.023**
FR	r					1	0.582	0.521	0.115	-0.094
	p-value						**<0.001**	**<0.001**	0.161	0.255
EF	r						1	0.291	-0.044	0.255
	p-value							**<0.001**	0.593	**0.002**
EOE	r							1	0.044	-0.121
	p-value								0.592	0.140
DD	r								1	-0.014
	p-value									0.869
CFNS	r									1
	p-value									

Subscales of the Child Eating Behavior Questionnaire - SR: satiety responsiveness; SE: slowness in eating; FF: food fussiness; EUE: emotional undereating; FR: food responsiveness; EF: enjoyment of food; EOE: emotional overeating; DD: desire to drink. CFNS: Child Food Neophobia Scale; r = Pearson’s Correlation Coefficient.

With regard to “slowness in eating,” there was a positive correlation with “emotional undereating” (p=0.042), and a negative correlation with “food responsiveness” (p=0.002) and “enjoyment of food” (p=0.000). “Food fussiness” negatively correlated with “enjoyment of food” (p=0.012) and CFNS (p=0.000). As for “emotional undereating,” a positive correlation with “emotional overeating” (p=0.004), and a negative correlation with CFNS scores (p=0.023) were observed. “Food responsiveness” positively correlated with “enjoyment of food” (p=0.000) and “emotional overeating” (p=0.000). Yet, “enjoyment of food” positively correlated with “emotional overeating” (p=0.000) and CFNS (p=0.002) as well.

## DISCUSSION

The study indicated that only a few domains of eating behavior significantly differed between age groups, with a tendency for higher score in “interest in food” on the part of older children, and in “lack of interest in food” on the part of younger children. Regarding the BMI-for-age classification, there was a tendency for higher scores in “food responsiveness” and “emotional overeating” by participants with severe obesity compared with those with normal weight, and no differences in relation to sex were observed. CFNS did not present significant differences in relation to age group, BMI-for-age, and sex. Overall, subscales related to interest in food positively correlated between each other, and negatively correlated with those related to lack of interest in food.

The subscales “slowness in eating” and “satiety responsiveness” tended to higher scores on the part of younger children. This datum is in agreement with the findings of Viana et al.^5^ and Wardle et al.,^7^ who pointed out in their studies tendencies for the scores of these subscales to increase with age. In the present research, older children tended to higher scores for interest in food (“food responsiveness,” “enjoyment of food,” and “emotional overeating”), which can also be observed in other studies.^5^ ^,^ ^7^

It is worth defining that “slowness in eating” is associated with lack of interest in food, whereas “satiety responsiveness” assesses the counterregulatory eating capacity, i.e., the regulation of appetite,^5^ factors that include intuitive eating. Intuitive eating can be defined as food intake in response to internal signs of hunger and satiety, rather than in response to emotions or external factors.^18^ Intuitive eating most frequently occurs in younger children, indicating an innate characteristic of the individual.

In a study on school-age children, the authors inferred that the CEBQ subscale “satiety responsiveness” can be an effective indicator of characteristics of response to internal signs of satiety, and “food responsiveness” and “enjoyment of food” are effective indicators of the capacity to respond to external food stimuli.^12^ This last finding is reinforced when considering the characteristics of these subscales, since “food responsiveness” refers to external food cues, such as flavor, color, aroma, which, together with “enjoyment of food,” assess the interest in food.^5^ Thus, it is observed that younger children tend to show greater lack of interest in food and greater response to their internal signs of satiety; on the other hand, older children have their eating behavior more influenced by external factors, which was also verified in the present study.

The category “interest in food” (“food responsiveness” and “emotional overeating”) tended to be higher in children with severe obesity. Studies indicate a positive relation between BMI and the subscales “food responsiveness,” “emotional overeating,”^10^ and “enjoyment of food.”^5^ ^,^ ^2^ In this respect, authors of some theories relate eating behavior to overweight, for example, the psychosomatic theory and the externality theory. The first highlights the influence of emotions on food intake, which, in overweight people, may be excessive in response to emotions, such as anger, fear, or anxiety, acting as a coping mechanism.^19^ Conversely, according to the externality theory, overweight people are more sensitive to external stimuli to eating behavior, such as sight, taste, and smell of foods,^20^ which could also be observed in older children of the present study.

None of the scales used in this research showed significant differences in relation to sex, corroborating data found in similar studies.^10^ ^,^ ^12^ ^,^ ^13^ ^,^ ^14^ ^,^ ^15^ However, other authors report that “slowness in eating”^5^ ^,^ ^3^ and “satiety responsiveness”^3^ were higher in girls, and “food fussiness” was higher in boys, highlighting the greater concern with weight regulation and feeding habits in females.

CFNS scores negatively correlated with “satiety responsiveness,” “food fussiness,” and “emotional undereating,” indicating that the higher the scores of these subscales, the lower the CFNS score, i.e., the more severe the food neophobia. These CEBQ subscales correspond to the lack of interest in food. In this sense, it can be considered that this characteristic is also present in food neophobia, considering that it includes resistance to eating and trying new foods.^9^ Studies reinforce that children with food neophobia present more food fussiness, less food preferences and consumption of fruits and vegetables.^14^ ^,^ ^15^ ^,^ ^16^ On the other hand, CFNS scores positively correlated with “enjoyment of food,” demonstrating that the higher the scores of this subscale, the higher the CFNS score, i.e., the more moderate the food neophobia. This result was also expected, considering that the enjoyment of food indicates interest in food,^7^ ^,^ ^5^ which is opposed to the aforementioned food neophobia characteristics.^9^

This study is one of the first to relate food neophobia to infant eating behavior in the Brazilian population. Nevertheless, it has some limitations that must be considered when interpreting the results, such as the cross-sectional study design, which does not allow confirming a causal relationship between the variables, the convenience sample, and the small sample size. Despite the limitations, the study indicated differences in some domains of the eating behavior between children of different ages and BMI-for-age, but not in relation to sex. Although no differences were found regarding food neophobia, overall, it positively correlated with lack of interest in food, and negatively correlated with interest in food.

Understanding how eating behavior is displayed at different ages, sexes, and nutritional statuses contributes to the implementation of nutritional interventions, seeking to assist children and adolescents in learning healthy behaviors according to each demand. Furthermore, understanding the relationship between eating behavior and food neophobia enables studying strategies that can be used by parents and caregivers without including persuasion, coercion, bribery, or the use of rewards to getting children to try new foods.
